# Managing Expectations and Predicting Willingness to Pay in Novel Healthy Foods Development in East Africa

**DOI:** 10.3390/foods14071258

**Published:** 2025-04-03

**Authors:** Alexander Mirzaei-Fard, Jesper Clement, John H. Muyonga, Olivia Janet Natocho, Josephine Kisakye, Susan Nchimbi-Msolla, Rashid Suleiman, Fulgence Mishili, Dasel Wambua Mulwa Kaindi, Sophia Ngala

**Affiliations:** 1 Department of Marketing, Copenhagen Business School, 2000 Frederiksberg, Denmark; jc.marktg@cbs.dk; 2 Department of Food Technology and Nutrition, Makerere University, Kampala P.O. Box 7062, Uganda; john.muyonga@mak.ac.ug (J.H.M.); janet.natocho@students.mak.ac.ug (O.J.N.); kisakyejk@gmail.com (J.K.); 3 Department of Crop Science and Horticulture, Sokoine University of Agriculture, Morogoro 3005, Tanzania; nchimbi@sua.ac.tz (S.N.-M.); rashid@sua.ac.tz (R.S.); fmishili@sua.ac.tz (F.M.); 4 Department of Food Science, Nutrition and Technology, University of Nairobi, Nairobi P.O. Box 29053, Kenyasngala@uonbi.ac.ke (S.N.)

**Keywords:** willingness to pay, packaging design, sensory evaluation, healthy foods, East Africa, consumer expectations, locality, xenocentrism

## Abstract

This study explores the factors influencing consumer willingness to pay (WTP) for novel, healthy, and locally produced food products in East Africa, focusing on sensory experiences and packaging design. Conducted in Tanzania, Uganda, and Kenya, the research includes two complementary studies: Study A examines sensory evaluations (taste, texture, aroma, color, and general acceptance) as predictors of WTP, while Study B assesses the impact of visual packaging features (e.g., typography, illustrations, and product windows) on consumer perceptions and WTP. Study A highlights that general acceptance (GA) is the strongest predictor of WTP, driven primarily by taste, texture, and aroma, while visual sensory cues play a secondary role. In contrast, Study B demonstrates that packaging design features, such as product visibility and ingredient-focused imagery, significantly influence WTP, with health messaging increasing perceived value but locality cues reducing it, likely due to cultural biases against packaged local products. The results reveal a critical difference: WTP is more stable and predictable in sensory evaluations but more volatile in response to packaging designs, driven by consumer expectations. These findings underscore the importance of aligning sensory and visual attributes to understand consumer expectations and enhance WTP for innovative food products in emerging markets.

## 1. Introduction

Food systems are shaped by the interconnected relationships between farmers, food processors, retailers, and consumers, where societal values around food consumption emerge through these interactions within a cultural economy, with growing emphasis on health, environmental sustainability, and cultural influences in food choices [1]. Food systems face multiple challenges, with climate change emerging as a significant external threat, leading to disruptions such as displacement and drought, which have profound societal consequences. Tackling these challenges demands innovative solutions and collaborative efforts from stakeholders across the system [2]. An increase in economic prosperity among consumers often leads to changes in dietary patterns, which in turn pose challenges to public health, as seen in many African countries [3]. The continent has experienced an increase in prosperity and wealth, but malnutrition continues to be a persistent issue, underscoring the need to explore food innovation within an African context [4]. However, research on consumers’ perceptions of food quality and their willingness to pay (WTP) for processed and packed foods in African countries remains limited [5].

The African food market is characterized by a combination of traditional small shops, kiosks, and large supermarkets, with an increasing share of processed and packaged food [6]. Re-thinking food supply involves engaging stakeholders, including farmers, industry, and crucially, the end consumers who will ultimately bear the cost of new food products and discern the success of the product. This process requires careful consideration of how to address and challenge consumers’ habitual behavior [7].

The price a consumer is willing to pay for a food product is closely tied to their subjective perception of its quality. From the producers’ perspective, the price is more objectively determined by factors such as raw material costs, production expenses, logistics, and marketing, which may not always align with the consumer’s perceived quality. Innovation and change in a food system inherently involve managing uncertainty surrounding the gap between consumer perceptions of quality and the pricing strategy for the new food product [8]. The aim of this article is to assess consumer willingness to pay (WTP) for new, healthy, packed, and locally produced food in Tanzania, Kenya, and Uganda and provide insights for developing effective marketing strategies for innovative healthy food products in East Africa.

## 2. Theoretical Background

For healthy packaged food products, the interpretation and initial decision to purchase are influenced by the packaging itself [9]. A recent study found that the visual impression of the packaging for the healthy and bitter virgin olive oil with a high content of polyphenols overshadows the taste experience and significantly increases WTP among consumers in Morocco and Tunisia [10]. The study also revealed that WTP increased when consumers were informed about the olive oil’s characteristics, such as origin and health benefits, which are often information given on the packaging. This underlines the added value in evaluating food packaging design as a distinct entity for the innovation process, where visually interpretable and relevant statements about the product contribute to the consumer’s overall perception of quality and experience, separate from the sensory attributes of the food itself. However, for new and innovative food products, a key unanswered question remains: How do consumers visually perceive and sensory evaluate healthy, innovative, and local food products, and to what extent does packaging design influence the price they are willing to pay for these unfamiliar offerings?

Cultural norms, social rules, local habits, and consumers’ attitudes towards healthy food play a significant role in guiding consumers’ decisions about what is considered right to eat or serve at home [11]. From a theoretical perspective, this behavioral process is often seen as stable over time, although research suggests that real-life food preferences may not be as stable as expected or directly applicable in consumer behavior [12]. The alignment between attitudes, preferences, and intention to purchase is particularly challenged when new food products are launched on the market [13], as this creates an uncertainty from unfamiliarity that may lead to skepticism toward the new food product.

When new food products create uncertainty towards the norms, consumers often evaluate a potential purchase based on how much it might disrupt what is important to them [14]. However, there is often a disconnect between explicit attitudes and what people consciously perceive as good or socially desirable and their actual purchasing behavior [12]. This misalignment can be attributed to the divergence between explicit and implicit attitudes. While explicit attitudes are influenced by social desirability and normative pressures, implicit attitudes are more tied to actual behaviors and are less affected by these external influences [15]. For instance, although consumers may explicitly express a preference for healthy food products due to perceived social acceptance, their implicit attitudes are rooted in taste preferences and previous experiences.

**Figure 1:**
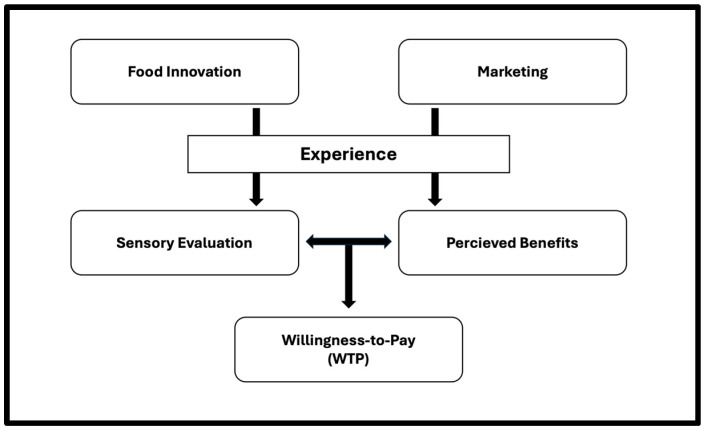
Schematic flowchart inspired by Deliza and MacFie [19].

Grasping implicit attitudes is crucial for predicting the success of an innovative healthy food product. Visually interpreting food packaging creates expectations, and later sensory experience determines whether these expectations are met [16]. This relationship is crucial for building trust and acceptance for new food products, as discrepancies between the sensory experience of food and the information or prior experience of the consumer will lead to reduced product acceptance [17]. For instance, taste plays a key role in shaping the overall evaluation of food quality, acting as a validation of the claims conveyed by the packaging [18]. Research indicates that discrepancies between expectation and sensory experience will lead to disconfirmation, affecting both satisfaction and future purchase behavior [19]. Therefore, understanding how sensory attributes match the visual interpretation from both the food and its packaging is essential for bridging consumer expectations and WTP. This study builds upon the large body of research within consumer experience and satisfaction, particularly well-versed expectation frameworks. It draws inspiration from the consumer expectation methodology outlined in the review by Deliza and MacFie [19] and the subsequent application of marketing variables [20] (Figure 1).

**Figure 2:**
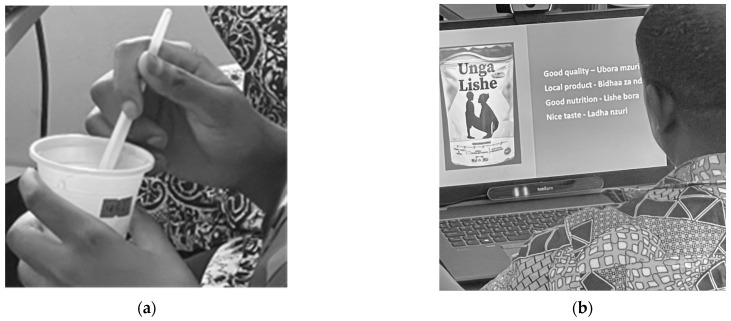
(**a**) Study A—tasting food samples; (**b**) Study B—interpretation of packaging.

The framework explores the role of visual stimuli from packaging design and messaging cues in shaping sensory evaluations, consumer attitudes, and general acceptance (GA). To investigate these factors influencing consumers’ WTP for novel healthy food products, two complementary studies were designed and conducted (Figure 2): one focusing on sensory evaluation (Study A) and a second focusing on visual perception of product packaging (Study B).

## 3. Research Design

**Figure 3:**
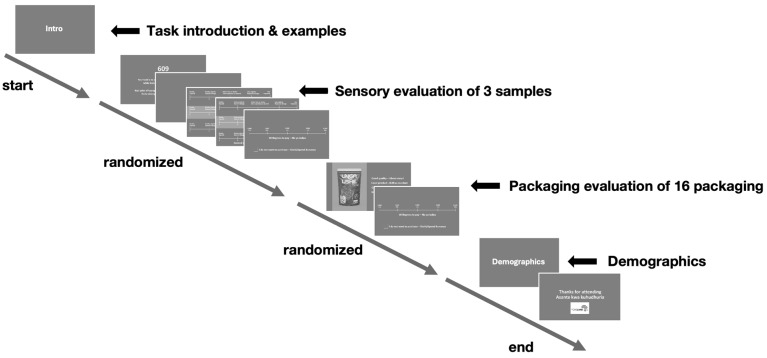
Protocol for the studies.

The study aims to bring new insights within an East African context related to the FoodLAND project [21], and data collection followed the same protocol for each of the three countries (Figure 3). Respondents were tested individually in controlled conditions and exposed in random order to the food sample in study A and the visual stimuli of packaging in study B. The taste test was conducted before the packaging test to avoid biasing the taste test.

In study A, samples were labeled with a random 3-digit label and given to respondents by operators when prompted on the screen. Each of the universities developed novel foods with alternate compositions to market standards. Uganda sampled daddies, traditionally a fried biscuit, but in this cake baked and made from composite flours of wheat, amaranth, and corn. Kenya sampled 3 compositions of tamarillo juice, plain, mixed with lemonade, and mixed with mulberry leaf extract, and three cookies made from composite flour and different 3 levels of quinoa. Tanzania sampled 6 types of porridge; (3 ready-to-eat and 3 that need cooking), each with varied composition. Respondents were recruited from university databases in Tanzania, Uganda, and Kenya to get a total sample size of 600+ described as urban consumers responsible for their household. In Tanzania, 200 respondents (166 f, 44 m) were tested on six porridges and 16 related designs of packaging that included different visual features (types of illustrations and typography). In Uganda, a body of 203 respondents (97 f, 106 m) were tested on dried eggplant and daddies (a crouton-shaped biscuit local to Uganda) and related 2 × 8 packaging designs, including the visual packaging feature enabling a view of the product inside (product window). In Kenya, 200 respondents (138 f, 61 m, 1 o) were tested on tamarillo juice and quinoa cookies, and 2 × 8 packaging designs featuring different shapes of packaging (bottle/carton/bag). These different packaging features enable us to investigate differences in the visual interpretation and their impact on WTP. Respondents are first exposed to a packaging design for 6 s, after which they choose one of four statements (health, nutrition, locality and taste) that best describe their perceived benefit of the product, before rating their WTP.

Analysis in A and B uses z-normalized WTP as the dependent variable, enabling cross-product and country comparisons by controlling for differences in scale biases related to specific product categories. Linear mixed-effects models are used to model WTP and GA, accounting for the nested structure of the data and random intercepts for respondents.

## 4. Study A—Sensory Testing of Novel Healthy Foods

### 4.1. Linking Expectations to Taste Evaluation

Research has shown that the color and appearance of a food product play a crucial role in the visual sensory processing and set expectations for its taste, especially for the intensity and expected flavor [22]. It has also been found that a dissonance between food color expectations and actual color leads to perceived differences in taste [23]. In addition, color serves as a cue for a food’s nutritional value and edibility [24]. Similarly, the smells and aromas function as olfactory indicators for food’s freshness, often complementing visual cues such as color [25], and by that, the taste is closely interconnected with olfactory cues, which can enhance or alter flavor perception. Texture, on the other hand, contributes to mouthfeel and the overall sensory experience, influencing consumer preferences and driving product acceptability [23,25].

### 4.2. Scales Used in Study A

Respondents tasted and evaluated 3–4 variations in a new food product equal in size and quantity across all participants, and each new food product was designed with healthier ingredients or reduced unhealthy processing compared to conventional products that were on the local market. Respondents rated each variation on 5-point Likert scales [26], related to five sensory variables: aroma, appearance, color, taste, texture, and lastly for general acceptance (GA).

Following these sensory ratings, respondents assess the WTP for a consistent product sample in local currency. WTP is measured on a 5-point scale with values determined by product category standards to give a price range, which is z-standardized within the product category to reduce scale bias and analyzed using linear mixed modeling to account for individual differences and repeated measures. To maintain comparability between the taste test and the later packaging test in study B for WTP, respondents are anchored with a blank package representing the size and quantity of the similar product packaging in study B.

### 4.3. Findings from Study A

GA emerges as the strongest predictor of WTP in the direct sensory evaluation model, underscoring acceptance as a central mechanism through which sensory attributes influence purchase intent for novel healthy food products. Table 1 and Table 2 show the results of analyzing the pathways of sensory variable influence using linear mixed effects modeling with respondents and product|countries as random effects. The estimated values are the models estimation of an incremental increase in predictor variables effect on GA and WTP.

General acceptance (GA) is significantly predicted by all five sensory variables: taste, texture, appearance, aroma, and color. Among these, taste is the most influential driver of GA (0.50). That is, taste can explain about half of the variation in GA and demonstrate its core role in shaping consumer perceptions of product ability to meet expectations. Moreover, texture, appearance, aroma, and color also contribute significantly to GA, albeit to varying degrees. This supports the notion that consumers consider multiple sensory cues when forming an overall judgment of the product (Table 1). Each sensory variable provides unique explanatory value to the model, indicating their collective importance in shaping GA. However, improving taste and texture is likely to yield the most pronounced impact on GA and reflect respondents’ holistic evaluation of the product’s sensory attributes.

In contrast, WTP reflects consumers’ final value interpretation of the product, showing a different weighting of sensory variables (Table 2). While taste remains the strongest predictor of WTP, its relative importance decreases (0.18) compared to its impact on GA. In plain words, taste has less importance when the consumer must assess how much the food should cost. Other sensory variables, such as appearance, aroma, and texture, gain a bit more relative prominence in influencing WTP. Interestingly, color, which was significant for GA, is not a significant predictor of WTP, indicating its limited role in driving consumers’ monetary valuation of the product in this context.

GA emerged as the strongest predictor of WTP when included alongside sensory variables, showing no signs of collinearity and acting as an independent construct rather than a compound of sensory attributes. This independence justifies its role as a mediating variable, capturing a holistic assessment that integrates sensory cues into purchase intent. Mediation analysis further underscores GA’s critical role, as the direct effects of sensory variables on WTP diminish when GA is included, highlighting its importance in translating sensory perceptions into purchase intent (Table 3).

The direct effects of color and taste are insignificant, having most of the variance in the model attributed to the mediated path, while aroma, texture, and appearance remain significant in their direct influence on WTP. Taste has the largest observed total effect on WTP and remains a critical factor for GA, although its influence on WTP becomes comparable to the effects of other sensory variables when the indirect pathway through GA is considered. Specifically, the combined mediated and direct effects show that taste aligns with the contributions of aroma and texture, while appearance has a smaller effect. As observed in Table 1 (GA) and Table 2 (WTP), much of the effect of taste and color on WTP is mediated through GA, leaving the observed direct effects of color insignificant, as found in Table 2.

### 4.4. Conclusions from Study A

When sampling novel healthy food products, acceptance is the most crucial factor in shaping consumer preferences and WTP, acting as both a predictor and a mediator in the relationships between sensory variables and purchase intent. The findings highlight that all five sensory variables—taste, texture, appearance, aroma, and color—contribute significantly to GA, with taste demonstrating the strongest influence (0.54). This underscores the centrality of taste in shaping product acceptance. However, as taste strongly influences acceptance, the direct effects of aroma and texture remain highly significant and impactful. Thus, the visual appearance of the food products, while expectation-inducing, appears to have a limited influence on purchase intentions. Instead, the sensory experiences that occur during consumption, such as the smell, mouthfeel, and taste of the product, are the critical direct and indirect drivers of price for novel foods. These findings emphasize the importance of managing expectations related to aroma, texture, and flavor during product development, while aspects like shape and color may play a less pivotal role.

Specifically, appearance, color, and aroma function as anticipation cues, setting initial expectations about the product’s quality and appeal and, in turn, acceptance and satisfaction. In contrast, taste and texture serve as confirmation cues, primarily reinforcing or adjusting these expectations during consumption, with aroma playing a dual role by bridging the gap between setting and confirming expectations. This distinction aligns with the dopamine reward prediction error (DRPE) model [27], emphasizing the dynamic interplay between sensory attributes and expectations in shaping consumer responses and enhancing product appeal [28].

### 4.5. Limitations in Study A

The mediation analysis revealed that GA serves as a critical mechanism through which sensory variables impact WTP, leveling the effects of individual variables. While taste remains pivotal for GA, its combined mediated and direct effect on WTP becomes comparable to the contributions of appearance, aroma, and texture. This suggests that GA operates as a distinct construct, influenced by sensory attributes, that independently predicts purchase intent for novel foods. Further research should aim to increase the variety of variables with existing literature affecting product acceptance. The mediated effects of both taste and color are among the highest, highlighting their reliance on GA to drive WTP. The products in this study varied very little in color, and the slight variations potentially contribute to the smaller effects observed in color as opposed to appearance variables.

Despite the robustness of these findings, several limitations need to be addressed to refine the proposed framework. For instance, attributes such as aroma and texture exhibit dual roles as both anticipation and confirmation cues. Aroma, for instance, contributes to taste perception while also shaping initial expectations, and texture influences both tactile expectations and weight and feel in hand and mouthfeel during consumption. This overlap complicates the clear categorization of sensory attributes, highlighting the need for further research to disentangle these dual functions. The framework also assumes a fixed sequence of sensory engagement (e.g., visual → olfactory → gustatory). However, deviations from this sequence or simultaneous sensory interactions may influence GA and purchase intent in unique ways. Investigating how alternative sequences or overlapping sensory inputs affect GA could provide deeper insights into the dynamic interplay of sensory attributes and the consumers’ WTP.

## 5. Study B—Visual Testing of Packaging Designs

### 5.1. Viewing Novel Food Packaging and Building up Expectation

Study B investigates East African urban consumers’ response to packaged food products, differentiating from rural market products where products are typically sold unpackaged on marketplaces or along roadsides. A specific interest is placed on marketing statements that can relate the new food product to a locality as a country-of-origin effect. The study explores specific visual packaging features designed to signal localness, healthiness, and quality by systematically manipulating these design features.

Participants were exposed to 16 variations in packaging and assessed their attitude toward one out of four statements related to the perceived benefit of the product: *Local Product, Good Nutrition (healthiness), Good Quality, and Nice Taste*. While nice taste serves as a control variable, the study focused on the impact of locality, healthiness, and quality cues on consumer expectations and their WTP. The visual packaging features tested were based on Deliza et al. [20] and included variations in packaging design features such as *color (few colors/colorful), illustration (people/ingredients), text information (healthy/local), typography (serif/sans-serif), ingredient list (horizontal/vertical), window (view to the product inside),* and *packaging shape (bottle/carton)*. Only four of these packaging design features were tested in one setup, allowing us to place the variations in a 4 × 4 matrix model and, by that, to isolate and analyze each feature parameter individually on the 16 different packages. Brand name and logo were omitted, as all food products were intended as new and unfamiliar to participants.

The study was carried out on an 18-inch laptop computer, starting with a task introduction, followed by a randomized slide deck of the 16 packaging stimuli, each displayed for 6 s to mitigate sequential bias. After viewing each stimulus, participants selected without any time limit one of the four statements as the best fit to their interpretation and lastly indicated their WTP on a continuous 5-point scale. Responses to statements were encoded as binary variables (yes/no), enabling logistic regression and mediation analyses. WTP data was z-standardized within product categories to ensure meaningful comparisons, mitigating scale bias deriving from product category price discrepancies and national currency variations.

### 5.2. Findings from Study B

**Figure 4:**
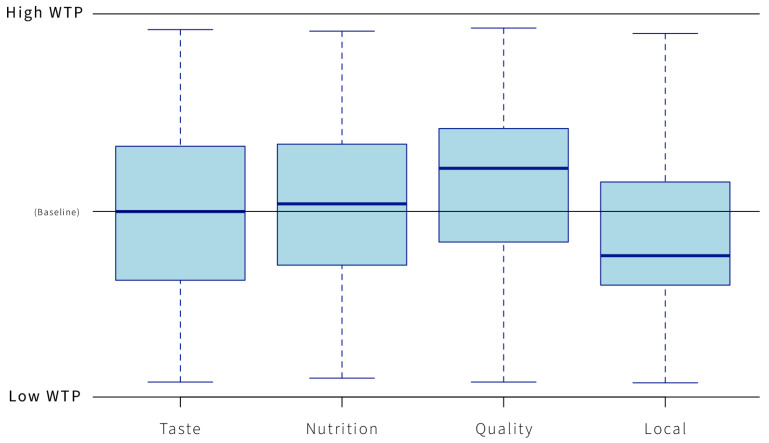
Comparison of statements on z-standardized WTP. WTP values are standardized within the mean of the products to enable comparison and reduce scale bias. The effect of packaging design features on WTP is outlined in Table 4, including the significance levels and the food product categories tested. Among the packaging features, illustrations of people had the largest impact on WTP when compared to packaging with illustrations of ingredients (*t*-value −11.73, *p*-value < 0.001). In addition, text information with serif typography was found to decrease WTP (*t*-value −3.32, *p*-value < 0.001), emphasizing the importance of using more modern, geometric, and curvier typography [29]. Furthermore, the placement of the ingredient list as a vertical table rather than plain horizontal text on the front of the packaging significantly increased WTP (*t*-value −0.13, *p*-value < 0.001). Including a view of the product in the packaging design (window) also led to a higher WTP (*t*-value 0.18, *p*-value < 0.001). This effect further correlated with the quality classification in statements provided.

Consumers’ perception of a product’s locality, healthiness, or quality had a significant and substantial impact on their WTP. When nice taste was used as a baseline, packaging that signaled good quality or healthiness/good nutrition increased WTP. However, when the packaging indicated localness, WTP decreased (Figure 4).

The relation between packaging design features and the four statements shows a significant level (Chi^2^ *p* < 0.001) for health information. Further, the text information about healthiness shapes consumer expectations and shows a significant increase in WTP and the likelihood that respondents will rate the products as having good nutrition, compared to text information emphasizing localness.

### 5.3. Conclusion on Study B

The information conveyed through product packaging directly influences WTP and indirectly influences the attitude toward the product. Among the various perceived product benefits, factors such as healthiness, locality, taste, or quality have the highest impact on WTP. Packaging that combines health-related symbols, clear text statements about health or locality, and visual cues effectively alters consumer perceptions, thereby impacting WTP. In the context of East African countries, symbols of locality such as country flags, messaging, and imagery/symbolism effectively reduce WTP. Moreover, packaging imagery that includes people compared to illustrations of ingredients will decrease WTP and should be avoided in the design of new food product packaging. In contrast, including a window to see the product inside the packaging has a positive effect and increases WTP.

## 6. Conclusions

We find a significant difference in our test persons’ WTP when they taste the product in study A and when they see the packaging tests in study B. WTP was significantly higher when people saw the packaging compared to the taste test (*p* < 0.001), and this was the case for each of the tested product categories. This finding is in line with a study conducted in Morocco and Tunisia [10], showing that WTP increases significantly when respondents are exposed to the packaging design. More information about the food product can increase WTP, and in this study, an ingredient list designed with a sans-serif typography increased WTP. Packaging design features like illustrations of ingredients and placing a window that allows people to see the product have the strongest impact on WTP. This was found across various categories, including cookies, daddies, juice, and porridge.

This study has a specific focus on new healthy food products that have not been launched on the market. The uncertainty that may arise in a consumer can be related to a discrepancy between expectations and actual experience [19]. This might give rise to a more skeptical sensory experience and, by that, a lower WTP, compared to looking at potential new packaging and being willing to pay for a well-designed packaging. Although showing a lower WTP when tasting the new food products, the data showed a smaller deviation in WTP and, by that, a higher degree of certainty in sensory preferences. The low variability of WTP in the taste study indicates that while price is more volatile for packaging, the stability of price perception in tasting is important for developing new healthy food products. Thus, managing general acceptance becomes the best pathway to change WTP, in which taste has the highest influence, while factors such as aroma and texture have a more direct influence on WTP.

Being exposed to different packaging designs exhibited greater variability in WTP, indicating that consumer expectations for new food products are more volatile and modulated by the visual interpretation of packaging design compared to the more consistent impact of actual sensory experience during the taste test. Although these findings do not correspond to findings from studies from Europe and the US [30], they underline the importance of designing and testing packaging for new food products specifically made for the East African market.

Testing new innovative food products at a University Campus can influence the test persons’ responses, and similarly, reflection can be placed on sample size and gender distribution in favor of women. Although research shows that women, specifically in African south-Sahara countries, tend to have the final say on food to be cooked [31], it calls for further research with a representative test panel.

## 7. Implications and Discussion

Packaging design features that indicate localness on product packaging, although associated with authenticity and cultural relevance, had a negative influence on WTP in this test. This may reflect a xenocentric bias, where consumers perceive local food products as less valuable when sold as packaged food. Traditional associations of local products with unpackaged goods sold in markets might exacerbate this perception. While packaging with local symbols and slogans did not directly affect WTP, it significantly influenced how the product was categorized, particularly as a “local product” which inherently contrasts with these expectations. On its own, packaging designed with local symbols like flags or text like slogans did not impact WTP, but these visual features had a significant relation to the statement of local and being classified as such. Interestingly, this negative impact from labeling localness on WTP contrasts with its potential to influence European consumers’ perceptions of quality. However, this aligns with consumer behavior observed in North and East African countries, where local products in certain ways may face resistance. Future studies should explore the potential negative attitudes toward local products among African consumers. Retailers and marketers should carefully evaluate the inclusion of locality features in product packaging design.

Certain packaging features, such as transparent windows, effectively signal product quality and align visual expectations with the sensory experience. However, use of packaging color had no measurable effect on WTP, indicating that packaging color alone is insufficient to drive purchase intention. Additionally, the use of sans-serif typography slightly increased WTP, suggesting a preference for modern packaging designs. How to make packaging more contemporary and modern calls for further research.

## Figures and Tables

**Table 1:** Sensory variables contribution to GA.

Sensory Variable	Effect on GA (Estimate ± SE)	*p*-Value
Taste	0.50 ± 0.016	<0.001
Texture	0.14 ± 0.017	<0.001
Appearance	0.12 ± 0.018	<0.001
Aroma	0.10 ± 0.018	<0.001
Color	0.09 ± 0.019	<0.001

**Table 2:** Sensory variables contribution on WTP.

Sensory Variable	Effect on WTP (Estimate ± SE)	*p*-Value
Taste	0.18 ± 0.021	<0.001
Appearance	0.13 ± 0.022	<0.001
Aroma	0.09 ± 0.023	<0.001
Texture	0.09 ± 0.021	<0.001
Color	0.01 ± 0.023	0.750

**Table 3:** Mediation of sensory variables on WTP through GA.

Variable	Total Effect	ACME	ADE	Proportion Mediated (%)
Taste	0.121 ***	0.068 ***	0.053	56 ***
Aroma	0.111 ***	0.013 ***	0.098 ***	12 ***
Texture	0.104 ***	0.019 ***	0.084 ***	19 ***
Appearance	0.077 **	0.015 ***	0.061 *	20 *
Color	0.033	0.012 ***	0.021	27

(Significance: *** < 0.001, ** < 0.01, * < 0.05), ACME = Average Casual Mediation Effect, ADE = Average Direct Effect.

**Table 4:** The packaging design features impact on WTP.

Packaging Features	Estimate	SE	*t*-Value	*p*-Value	Products Tested
**Color** (few colors/colorful)	0.0019	0.0023	0.856	0.392	Cookie, eggplant, daddies
**Illustration** (people/ingredients)	−0.2083	0.0178	−11.73	<0.001	Porridge, Juice, Cookies
**Text information ** (healthy/local)	0.0041	0.0021	1.985	0.047	Cookie, Juice, Eggplant, Daddies
**Typography** (serif/sans-serif)	−0.0560	0.0168	−3.324	<0.001	Porridge, Eggplant, Daddies
**Ingredient list ** (horizontal/vertical)	−0.1333	0.0232	−5.737	<0.001	Porridge
**Window** (view to the product inside)	0.1847	0.0026	7.062	<0.001	Daddies, Eggplant
**Packaging shape ** (bottle/carton)	0.0764	0.0477	1.600	0.1100	Juice

## Data Availability

The original contributions presented in the study are included in the article, further inquiries can be directed to the corresponding author.

## Abbreviations

The following abbreviations are used in this manuscript:

WTP	Willingness to pay
GA	General acceptance
